# Effectiveness and safety of sirolimus in the treatment of venous malformations: A meta-analysis of prospective studies

**DOI:** 10.1016/j.jvsv.2025.102284

**Published:** 2025-07-05

**Authors:** Guoyong Wang, Wei Lu, Yingjie Zhu, Chaonan Wang, Xiaonan Yang

**Affiliations:** Department of Hemangioma and Vascular Malformation, Plastic Surgery Hospital, Chinese Academy of Medical Sciences and Peking Union Medical College, Beijing, China

**Keywords:** Meta-analysis, Prospective studies, Sirolimus, Vascular anomalies, Venous malformations

## Abstract

**Background:**

Venous malformations are prevalent vascular anomalies. Recent clinical studies have explored the use of sirolimus for these conditions, particularly in patients who are not candidates for, or have shown limited response to, traditional treatments such as sclerotherapy and surgery. This meta-analysis systematically evaluates the efficacy and safety of sirolimus in treating venous malformations.

**Methods:**

We conducted searches in PubMed, Cochrane Library, Web of Science, and the Cochrane Database of Systematic Reviews until August 10, 2024. The quality of the included prospective studies (including both randomized and single-arm pre-post trials) was assessed using the Cochrane risk of bias tool. Statistical analyses were performed with Review Manager (Version 5.4).

**Results:**

Our analysis included eight prospective studies involving 74 patients. Primary outcomes measured changes in the volume and size of the malformations. Secondary outcomes assessed were functional disability scores, hemoglobin levels, coagulation indices, transfusion requirements, patient quality of life, and radiologic responses. Sirolimus demonstrated significant therapeutic benefits, with an odds ratio of 0.02 (95% confidence interval, 0.00-0.08) across six studies evaluating dichotomous variables. Results for continuous variables were consistent. Sirolimus showed safety in short-to medium-term use, with reversible mild to moderate side effects such as oral ulcers and liver function abnormalities. No severe adverse events (grade 3-5) were reported.

**Conclusions:**

Sirolimus is effective and safe for treating venous malformations, especially in patients unresponsive to conventional therapies. Future studies should explore long-term effects, optimal dosages, and administration techniques.

Venous malformations represent the most prevalent type of congenital vascular anomalies.^1^ These malformations occur in approximately 1 to 2 per 10,000 newborns and are found in about 1% of the general population.^2^ They are characterized by structural irregularities, inflammatory processes, and genetic mutations. The venous walls in these malformations are notably weaker than normal, primarily due to diminished elastic fibers and smooth muscle cells, coupled with defects in connective tissue components.^3^ Inflammation plays a critical role, with macrophages accumulating in affected areas and exacerbating the malformation through the secretion of cytokines and growth factors.^4^ Genetic investigations have linked some venous malformations to specific mutations, notably in the TIE2 (TEK) gene.^5^ Sirolimus is commonly used as an immunosuppressant in organ transplantation and specific disease treatments.^6^ It inhibits the mammalian target of rapamycin (mTOR) pathway, a pivotal protein kinase regulating cell proliferation, survival, angiogenesis, and protein synthesis.^7^ Additionally, sirolimus possesses anti-angiogenic properties, inhibiting endothelial cell proliferation and migration, thereby preventing new blood vessel formation.^8^ It further mitigates inflammatory cell activation and cytokine release, thus aiding in managing the inflammatory response associated with venous malformations.^9^

Current treatment strategies for venous malformations include sclerotherapy, laser therapy, surgical resection, and compression therapy, each presenting specific limitations.^10^ Sclerotherapy can lead to side effects such as pain, swelling, and skin discoloration.^11^ Although laser therapy is effective for small, superficial venous malformations, it is less effective for deep or extensive ones and may result in skin burns or pigmentation.^12^ Surgical resection, although it can completely remove the malformation, poses increased risks in cases of extensive or structurally critical malformations.^13^ Compression therapy primarily helps alleviate symptoms in limbs but does not decrease malformation size or prevent its progression.^14^

Sirolimus is particularly effective for managing extensive or complex venous malformations that are not amenable to surgical or other physical interventions.^15^ Research indicates that sirolimus substantially alleviates pain and exudation in patients with mixed-type malformations. Furthermore, a prospective clinical trial demonstrated significant improvements in complex vascular anomalies and superficial slow-flow vascular malformations among children and adolescents treated with sirolimus.^16^ Consequently, this study aims to evaluate the efficacy and safety of sirolimus treatment for venous malformations through a meta-analysis.

## Methods

### Design

Systematic searches were conducted in electronic databases to identify articles discussing the treatment of venous malformations with sirolimus. This systematic review was designed according to the Preferred Reporting Items for Systematic Reviews and Meta-Analyses (PRISMA) guidelines,^17^ The completed PRISMA checklist is provided in the Supplementary Appendix (online only). A comprehensive literature review of published reviews was performed, employing the Joanna Briggs Institute (JBI) methodology for systematic review processes. The review and meta-analysis adhered strictly to the PRISMA statement. This study has been registered with the International Prospective Register of Systematic Reviews (PROSPERO) under the registration number CRD42024578128.

### Search strategy

The search strategy adopted the PICOS (Patient, Intervention, Comparison, Outcome/Study Design) framework. Systematic searches to assess the efficacy and safety of sirolimus in treating venous malformations were conducted from the inception of the study until August 10, 2024, across multiple databases including PubMed, Cochrane Library, Web of Science, and the Cochrane Database of Systematic Reviews. The most recent search in these databases occurred on August 10, 2024.The following search terms were used: (“sirolimus” [MeSH Terms] OR “sirolimus” [All Fields] OR “rapamycin” [MeSH Terms] OR “rapamycin” [All Fields] OR “mTOR inhibitor” [MeSH Terms] OR “mTOR inhibitor” [All Fields] OR “everolimus” [MeSH Terms] OR “everolimus” [All Fields] OR “temsirolimus” [MeSH Terms] OR “temsirolimus” [All Fields]) AND (“venous malformation” [MeSH Terms] OR “venous malformation” [All Fields] OR "vascular malformation" [MeSH Terms] OR “vascular malformation” [All Fields] OR "vascular anomal" [MeSH Terms] OR “vascular anomal” [All Fields]) AND ("clinical trial"[Publication Type] OR "randomized controlled trial"[Publication Type] OR "cohort study"[Publication Type] OR "case series"[Publication Type]).

### Selection of studies

**Fig 1:**
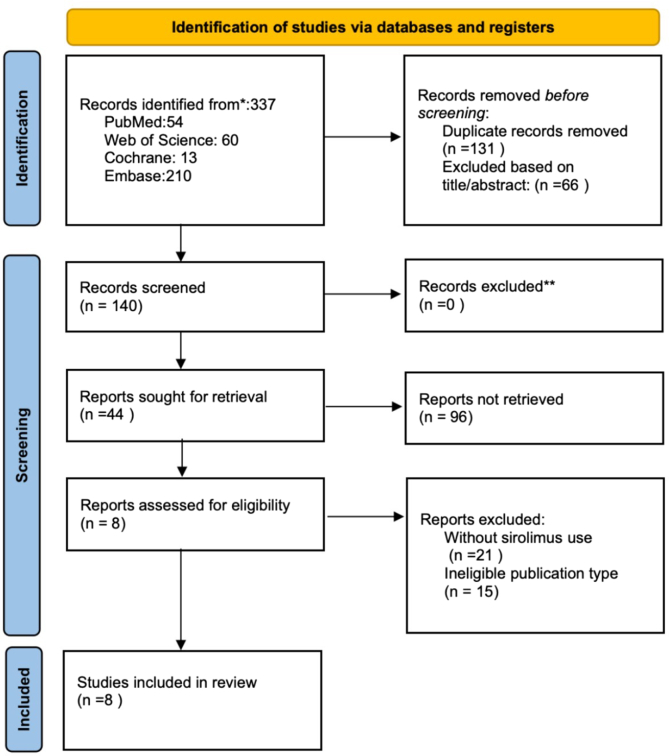
Flow chart of literature search results and details of included and excluded articles.

Initially, two researchers (WL and YZ) established the retrieval strategy aligned with the research objectives, removed duplicate literature, and then independently screened articles based on titles and abstracts to exclude irrelevant studies. Subsequently, they reviewed the full texts of the selected articles and independently identified those that met the inclusion and exclusion criteria, from which data were extracted. Each chosen study was assessed by two independent researchers (WL and YZ) who documented essential details such as the first author, publication year, sample size, and target population in a predefined table. Exclusions from the analysis included reviews, commentaries, conference papers, duplicate publications, and studies lacking essential information, research results, full-text availability, or those reliant on alternative tools for measuring outcomes. Discrepancies at any stage of the review were resolved through consultation, involving a third researcher (CW) when necessary. The screening process is depicted in Fig 1.

The study selection criteria were detailed as follows: Inclusion criteria encompassed all reports detailing the treatment of venous malformations with sirolimus (topical or oral), alone or in conjunction with other therapeutic drugs, across clinical trials, randomized controlled trials, cohort studies, and case series, with no restrictions on age or gender. Exclusions included duplicate publications, reports with insufficient information, and opinion and revision articles. Furthermore, the references of the selected articles were examined to comprehensively identify additional relevant literature. The primary efficacy endpoints were changes in the volume of vascular malformations as observed on magnetic resonance imaging (MRI), alterations in the size of venous malformation lesions, or clearance rates determined by colorimetry. Secondary outcomes encompassed functional impairment scores, hemoglobin levels, coagulation indicators, transfusion requirements, patient quality of life, and radiologic responses. The primary safety assessments were based on the incidence of adverse events.

Exclusion criteria: (1) Studies with outcomes of interest that may include other types of vascular malformations, such as capillary malformations, arteriovenous malformations, etc; (2) Studies not involving the relevant drug, sirolimus; (3) Case reports, reviews, conference abstracts, or posters; and (4) Non-human studies or studies not in English.

### Data extraction

Data were extracted into a Microsoft Excel spreadsheet database, encompassing publication metrics such as the first author’s name, year of publication, and country of origin. Included were details on study design, types of vascular anomalies classified according to the International Society for the Study of Vascular Anomalies, pathologic complications, patient demographics (gender, age, number of lesions, size), and treatment specifics (sirolimus dosage, blood levels, cointerventions, treatment duration, adverse reactions, efficacy). Additionally, criteria for clinical benefit and follow-up data were recorded.

### Statistical analysis

In these studies, dichotomous outcomes such as radiologic response and clinical improvement were expressed as proportions and analyzed using a binomial distribution to estimate pooled response rates. Heterogeneity among the studies was assessed using the I^2^ test, with values of 0.25, 0.50, and 0.75 indicating low, moderate, and high heterogeneity, respectively. A *P*-value of less than .05 was considered indicative of statistically significant heterogeneity. A random effects model was utilized to aggregate selected studies and estimate the percentage of scores. The risk of bias for all included prospective studies was evaluated using the Cochrane Collaboration’s risk assessment tool (https://handbook.cochrane.org), with appropriate adaptations for single-arm study designs where applicable. A random effects model using the DerSimonian–Laird method was applied to synthesize results across studies, given the expected clinical and methodological heterogeneity in study populations, interventions, and outcome definitions. This modeling choice was made a priori, independent of heterogeneity statistics. Forest plots were utilized to visually represent selected studies based on effect size and 95% confidence intervals (CIs), whereas funnel plots based on Egger’s regression test were used to assess the impact of small studies and potential publication bias. All analyses were conducted using Review Manager (Version 5.4).

## Results

### Study selection

The PRISMA flow chart for study selection is depicted in Fig 1. Initial searches in electronic databases yielded 337 unique studies. Upon review of titles and abstracts by two independent reviewers (WL and YZ), 44 studies were selected for full-text evaluation. Of these, 36 studies were subsequently excluded: 21 for irrelevant use of sirolimus and 15 due to mismatched study types. Ultimately, eight studies met the inclusion criteria for this systematic review and meta-analysis.^16^^,^^18^, ^19^, ^20^, ^21^, ^22^, ^23^, ^24^ The included studies encompassed 74 cases of venous malformations, including 11 cases of blue rubber bleb nevus syndrome (BRBNS), a rare genetic disorder characterized by multiple venous malformations primarily affecting the skin and gastrointestinal tract. Patients with BRBNS typically exhibit blue or purple skin lesions (blue rubber bleb nevi), resulting from abnormally dilated subdermal veins, as detailed in Table I.

**Table I:** Characteristics of included studies

Author	Year	Design	Sirolimus	Population	Country	No.	Outcomes used in meta-analysis	Type; assessment of outcomes
Annabel et al.	2021	Multi-center RCT	Oral, 4-12 ng/mL	Children (6-18 years old) with venous malformation	France	22	The primary outcome is the change in the volume of vascular malformations detected on MRI scans (interpreted in detail) per unit of time between the intervention period and the observation period.	Continuous variable; mean [standard deviation] 0.001 [0.004]
Denise M et al.	2016	Single-center RCT	Oral, starting at 0.8 mg/m^2^ per dose twice daily	Patients with venous malformation at Cincinnati Children’s Hospital Medical Center	Boston	12	Responsiveness to sirolimus (evaluated based on functional disability scores, quality of life, and radiologic assessment).	Dichotomous variable; Courses response: PR 11 NE 1
Yi Ji et al.	2021	Multi-center RCT	Oral, starting at 0.8 mg/m^2^ per dose twice daily	Patients with venous malformation	China	19	The response, which was measured using sequential volumetric MRI.	Dichotomous variable; GR 4 (21.1) PR 12 (63.2) SD 2 (10.5) PD 1 (5.3)
Veroniek E M et al.	2021	Single-center RCT	Oral, target levels ranging between 4 and 10 ng/mL	Patients with venous malformation	Netherlands	2	The response to sirolimus after 6/12 months was assessed using MRI to evaluate the size of vascular malformations.	Dichotomous variable; 6 months: Improvement of symptoms 100% Decrease 50% No MRI 50% 12 months: Pain-free 50% Not restarted 50% Decrease 50% Not restarted 50%
Mari et al.	2021	Single-center RCT	Oral, 0.2% sirolimus gel	Patients with venous malformation	Japan	2	The primary endpoint is the safety assessment of sirolimus gel. The main secondary endpoint is the improvement rate assessed by the Central Judging Committee using photographs at 12 weeks.	Dichotomous variable; No adverse events 100% Blood sirolimus concentration: Below the measurement limit Relief of pain 100%
Zhou J. et al.	2021	Two-center RCT	Oral, sirolimus at the dose of 1.0 mg/m^2^	BRBNS	China	11	The primary outcome is the change in the size of venous malformation lesions. Secondary outcomes include changes in Hb levels, coagulation indicators, transfusion requirements, patient quality of life, and adverse events.	Continuous variable; MRI assessment Statistical analysis yielded an F ratio of F(3,21) = 11.571; *P* < .001. Compared with baseline (19.0 ± 6.9 mm), the size of lesions decreased by 7.4% at month 3 (17.6 ± 5.6 mm; *P* = .004), by 9.3% at month 6 (17.2 ± 5.9 mm; *P*=.001), and by 13.0% at month 12 (16.5 ± 5.6 mm; *P* < .001). Dermatologic quality-of-life scores did not show improvement. Mean Hb levels: The first 84 ± 9 g/L; 86 ± 10 g/L (3 months) 108 ± 19 g/L (6 months) 107 ± 21 g/L (12 months). Reduced patient need for transfusion. D-dimer levels decreased in 8 of 10 patients (80%). Fibrinogen levels were slightly reduced in 5 participants (45%) before treatment. Improved quality of life for patients, with lighter stool color, improved physical strength, and better cheek color after treatment. Adverse events included oral ulcers, liver dysfunction, hypercholesterolemia, acne, and hair loss, with no grade 3-5 adverse events observed.
Jennifer et al.	2018	Single-center RCT	Oral. Children under the age of 12 years started with a dose of 0.8 mg/m^2^ body surface, twice-a-day; older patients with a single dose of 2 mg/day.	Symptomatic patients with venous malformation that were refractory to standard care, such as sclerotherapy and/or surgical resection	Belgium	4	The primary endpoint is the efficacy and safety of sirolimus at 12 months. The drug’s efficacy is evaluated based on physical (clinical size, color, and consistency of the lesion), functional (organ dysfunction, mobility impairment, pain, bleeding, exudation, recurrent infection), biological (measurement of coagulation parameters), and radiologic responses, as well as quality of life questionnaires.	Dichotomous variable; Physical examination 3/3; Organ or mobility dysfunction 4/4; Pain 4/4; Bleeding 1/1; Oozing 0/0; Repetitive infections 0/0; Quality of life: Moderate amelioration 1/4, Strong amelioration 3/4; Reduced coagulopathy 4/4; MRI: Progression 1/4, Status quo 2/4, Amelioration 1/4; ITK-SNAP 3–0: No volume modification 1/4, Volume reduction 3/4
A. Jerez et al.	2017	Single-center RCT	Oral. The initiation of a dose of sirolimus 2 mg per day	Patients with venous malformation	Venezuela	2	Assessment of venous malformation lesion size, color, texture, and pain.	Dichotomous variable; significant improvement was observed in all parameters (100%). No side effects caused by sirolimus were observed within 12 months.

*BRBNS,* Blue rubber bleb nevus syndrome; *GR,* good response; *Hb,* hemoglobin; *MRI,* magnetic resonance imaging; *NE,* not evaluable; *PR,* partial response; *RCT,* randomized controlled trial; *SD,* stable disease.

Data are presented as mean (standard deviation) or median (interquartile range).

### Efficacy

In eight articles, definitions of the efficacy of sirolimus treatment for venous malformations vary. Four articles primarily assess efficacy by monitoring volume changes in vascular malformations via MRI, whereas three articles focus on changes in lesion size. Additionally, efficacy is evaluated based on functional disability scores, hemoglobin levels, coagulation indicators, transfusion requirements, patient quality of life, and radiologic responses.

**Fig 2:**
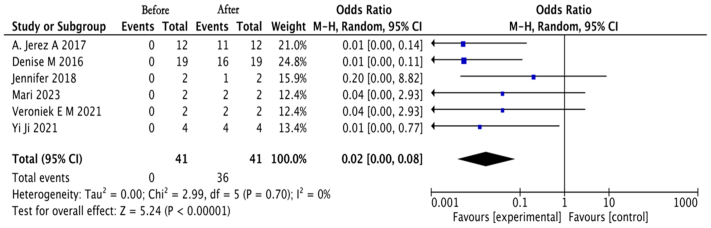
Forest plot before and after sirolimus treatment. ∗Combined estimates (represented by *black diamonds*) of overall weighted effects were calculated. *CI*, Confidence interval.

Among the eight studies included, six reported dichotomous outcomes based on pre- and post-treatment comparisons within the same patient group, whereas two addressed continuous variables. Due to substantial clinical heterogeneity across studies—including differences in patient characteristics, intervention types, and outcome assessments—all pooled analyses employed a random-effects model, which accounts for variation in true effect sizes between studies. The meta-analysis of the six studies with dichotomous outcomes (n = 41) yielded an odds ratio (OR) of 0.02 (95% CI, 0.00-0.08) (Fig 2). In one of the articles addressing continuous variables, the primary outcome was the rate of change in vascular malformation volume as detected by MRI (interpreted centrally), which showed a decrease post-treatment. Another article by Zhou et al reported a reduction in the size of venous malformations as the primary outcome, with secondary outcomes including changes in hemoglobin levels, coagulation indicators, transfusion requirements, patient quality of life, and adverse events. MRI assessments confirmed these reductions. Despite these positive changes, the Dermatology Life Quality Index scores showed no significant improvement. Before treatment, patients exhibited anemia with an average hemoglobin level of 84 ± 9 g/L, which significantly increased post-treatment. Sirolimus also notably reduced the need for transfusions and led to partial normalization of D-dimer levels and some improvement in fibrinogen levels. Ultimately, sirolimus was associated with improved patient quality of life, evidenced by reports of lighter stool color, increased physical strength, and enhanced facial complexion.

### Safety

Eight articles evaluated the safety of sirolimus by monitoring changes in adverse events, including one study focused on the safety of a topical sirolimus gel. In research related to BRBNS, 81.8% of patients experienced grade 1 to 2 episodic oral ulcers, with most reporting relief within 1 to 2 months of treatment. Two patients (18.2%, grade 1-2) demonstrated transient increases in liver enzymes. Specifically, one patient showed a slight elevation in aspartate aminotransferase (43 U/L) during the third month, whereas another exhibited elevated alanine aminotransferase (198 U/L) and aspartate aminotransferase (67.1 U/L) levels in the first month; both cases normalized within 2 weeks without discontinuing sirolimus. After 1 year of treatment, triglyceride levels remained stable, although there was a moderate, yet normal range, increase in total cholesterol. Additionally, 27.3% of patients reported grade 1 acne, and 9.1% experienced grade 1 hair loss. No severe adverse events (grade 3-5) occurred. All parameters studied in pediatric venous malformations treated with sirolimus showed significant improvement, with no severe side effects reported during the 12-month study period.

### Quality of literature

**Fig 3:**
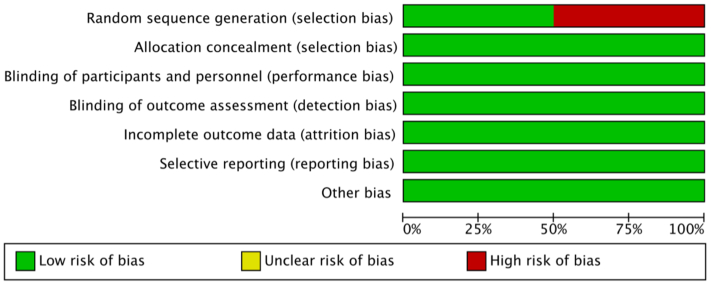
Risk of bias assessment and overall quality of included studies.

An assessment of bias was conducted on the eight included articles, revealing that four exhibited selection bias, primarily due to the small number of participants and their single-center design. The remaining four articles were of high quality and demonstrated low bias (Fig 3).

## Discussion

This paper reviews eight studies involving 74 patients with venous malformations to evaluate the efficacy and safety of sirolimus for treating these conditions. The findings indicate that sirolimus significantly reduces the volume of vascular malformations, enhances hemoglobin levels, and improves coagulation function, thereby diminishing the need for transfusions and substantially improving patients’ quality of life. Although mild to moderate adverse events such as oral ulcers and liver function abnormalities occurred, these were generally reversible upon discontinuation or dose reduction. Notably, no severe adverse events (grade 3-5) were reported, suggesting that sirolimus is safe for short- to medium-term use. All parameters studied in pediatric patients with venous malformations exhibited significant improvement over the 12-month study period, with no severe side effects attributed to sirolimus. However, it is important to note that several studies have reported a recurrence or rebound of venous malformations following sirolimus discontinuation, likely due to reactivation of the mTOR signaling pathway.^25^^,^^26^ This suggests that sirolimus may act as a disease-modifying rather than curative agent, and highlights the need for individualized treatment duration, long-term follow-up, and further investigation into optimal tapering or maintenance strategies. In addition, sirolimus may improve hematologic parameters associated with chronic disseminated intravascular coagulation (DIC)-like states in patients with extensive venous malformations. For example, Zhou et al reported that D-dimer levels decreased in 80% of patients, fibrinogen levels partially normalized, and hemoglobin concentrations increased after treatment, accompanied by reduced transfusion requirements.^22^ These findings suggest a broader role for sirolimus in managing both structural and hematologic complications in complex cases. Although the pooled OR of 0.02 appears numerically small, this reflects a high response rate and a very low event rate of treatment failure, indicating a strong and clinically meaningful therapeutic effect.

Our study affirms that sirolimus is an effective treatment for venous malformations, a finding supported by the research of Sandbank and colleagues. A multicenter and retrospective study reported that rapamycin is effective in 85% of vascular anomaly cases, achieving complete remission in five cases.^27^ Durán-Romero and others found that sirolimus, when used in patients with high-flow venous malformations, results in a partial response in 88.9% of cases and is generally well-tolerated.^28^ Similarly, a multicenter case series demonstrated that topical sirolimus application improves symptoms in most patients with vascular malformations.^29^ Yesil and others, through a single-center experience, observed that sirolimus is safe and effective, particularly in pediatric patients.^30^ Our findings corroborate that sirolimus is safe for short- to medium-term use in patients with venous malformations. Kim and colleagues documented that 20 patients, ranging from 1 month to 19 years old with various vascular malformations, showed good tolerance to sirolimus over an average of 2.1 years, with minimal complications aside from a case of *Pneumocystis jirovecii* pneumonia.^31^ A systematic review and meta-analysis suggest that sirolimus is a relatively safe treatment option for vascular malformations, with tolerable side effects.^32^

The limitations of this study are as follows: (1) The included studies are characterized by small sample sizes and are predominantly single-center, increasing the risk of selection bias and reducing the generalizability and reliability of the findings. In particular, some studies with extremely limited sample sizes may be subject to floor effects, potentially biasing effect size estimation and contributing to variability in the pooled outcomes. (2) There is considerable heterogeneity among the studies in terms of design, patient characteristics, intervention methods, and outcome assessment indices. Despite the use of a random-effects model for analysis, this heterogeneity could still compromise the robustness of the findings. (3) The dosage and administration routes of sirolimus varied across the studies, with the impact of these variations on the final outcomes remaining underexplored. Furthermore, most studies featured short follow-up periods, precluding a comprehensive assessment of the long-term effects and potential adverse reactions of sirolimus. (4) Publication bias may exist, as the exclusion of unpublished small-scale studies or studies reporting negative results could skew the comprehensiveness of the analysis.

The findings of this study have substantial implications for clinical practice. Sirolimus presents a viable alternative for patients who do not respond adequately to conventional treatments such as sclerotherapy or surgical removal, especially in complex cases. Moreover, sirolimus significantly ameliorates anemia and reduces the need for transfusions in patients with BRBNS. Considering its potential applications, sirolimus is poised to become an integral component of personalized treatment strategies. Its synergistic use with other therapeutic modalities, including surgery and sclerosing agents, could further improve therapeutic outcomes. For instance, sirolimus may reduce the size and vascularity of lesions, thereby allowing sclerotherapy to be performed more precisely and with lower volume, which could minimize procedural risks and treatment costs. In turn, successful sclerotherapy may enable discontinuation of sirolimus in selected cases, offering both clinical and economic benefits. This combined approach warrants further investigation in future studies. Future research should prioritize several areas: First, conducting large-scale, multicenter randomized controlled trials to mitigate the biases associated with small sample sizes and single-center studies. Additionally, it is crucial to extend evaluations to the long-term efficacy and safety of sirolimus, moving beyond the current emphasis on short-term effects. Finally, investigating the role of genomics and other biomarkers in predicting treatment responses is essential for advancing personalized treatment approaches.

## Conclusions

This study confirmed significant efficacy of sirolimus in treating venous malformations, effectively decreasing malformation size and enhancing patients’ quality of life. Despite mild to moderate adverse reactions like oral ulcers and liver function abnormalities, these side effects were generally reversible upon discontinuation or dose reduction, and no severe adverse events were recorded.

## Clinical recommendations

(1) For patients with venous malformations who have not benefited from traditional treatments such as sclerotherapy, laser therapy, or surgery, sirolimus should be considered as a primary treatment option.
(2) Regular monitoring of liver function and hematologic parameters is essential during sirolimus treatment, with adjustments to the dosage as necessary to minimize adverse reactions and maximize treatment efficacy.
(3) Considering the focus of current research on short-term efficacy and safety, clinicians are advised to monitor the long-term effects and potential risks associated with sirolimus treatment and ensure timely follow-up assessments.

## Author contributions

Conception and design: GW, XY

Analysis and interpretation: WL, CW

Data collection: GW, YZ

Writing the article: GW

Critical revision of the article: GW, WL, YZ, CW, XY

Final approval of the article: GW, WL, YZ, CW, XY

Statistical analysis: Not applicable

Obtained funding: Not applicable

Overall responsibility: XY

## Funding

This study was financially supported by the National Clinical Key Specialty Construction Project (23003), the Plastic Medicine Research Fund of 10.13039/501100005150Chinese Academy of Medical Sciences (2024-ZX-1-01), and the National Major Disease Multidisciplinary Diagnosis and Treatment Cooperation Project (No.1112320139). The funding body played no role in the design of the study and collection, analysis, and interpretation of data and in writing the manuscript.

## Disclosures

None.
